# Nutritional Systems Biology Modeling: From Molecular Mechanisms to Physiology

**DOI:** 10.1371/journal.pcbi.1000554

**Published:** 2009-11-26

**Authors:** Albert A. de Graaf, Andreas P. Freidig, Baukje De Roos, Neema Jamshidi, Matthias Heinemann, Johan A.C. Rullmann, Kevin D. Hall, Martin Adiels, Ben van Ommen

**Affiliations:** 1 Biosciences, TNO Quality of Life, Zeist, The Netherlands; 2 Amsterdam Molecular Therapeutics, Amsterdam, The Netherlands; 3 Rowett Research Institute, Aberdeen, United Kingdom; 4 Department of Bioengineering, University of California, San Diego, La Jolla, California, United States of America; 5 Institute of Molecular Systems Biology, ETH Zurich, Zurich, Switzerland; 6 Department of Molecular Design & Informatics, NV Organon, a part of Schering-Plough Corporation, Oss, The Netherlands; 7 Laboratory of Biological Modeling, NIDDK/NIH, Bethesda, Maryland, United States of America; 8 Sahlgrenska Academy at University Göteborg, Göteborg, Sweden; University of California San Diego, United States of America

## Abstract

The use of computational modeling and simulation has increased in many biological fields, but despite their potential these techniques are only marginally applied in nutritional sciences. Nevertheless, recent applications of modeling have been instrumental in answering important nutritional questions from the cellular up to the physiological levels. Capturing the complexity of today's important nutritional research questions poses a challenge for modeling to become truly integrative in the consideration and interpretation of experimental data at widely differing scales of space and time. In this review, we discuss a selection of available modeling approaches and applications relevant for nutrition. We then put these models into perspective by categorizing them according to their space and time domain. Through this categorization process, we identified a dearth of models that consider processes occurring between the microscopic and macroscopic scale. We propose a “middle-out” strategy to develop the required full-scale, multilevel computational models. Exhaustive and accurate phenotyping, the use of the virtual patient concept, and the development of biomarkers from “-omics” signatures are identified as key elements of a successful systems biology modeling approach in nutrition research—one that integrates physiological mechanisms and data at multiple space and time scales.

## Introduction

Nutritional science is presently undergoing a data explosion as an increasing number of studies are incorporating methods from genomics, transcriptomics, proteomics, and metabolomics. However, it is presently unclear how these high-dimensional datasets can be related to the physiological characterization of phenotype using traditional nutritional research methods such as indirect calorimetry, nutrient balance, body composition assessment, and isotopic tracer methods. Thus, a fundamental challenge for nutrition research is to connect these data that are collected at vastly different spatial, temporal, and dimensionality scales. Although statistical analysis is still the method of choice to deal with the high dimensionality of “-omics” datasets, systems biology and computational modeling approaches begin to reveal quantitative mechanistic relationships between these various measurements.

A large variety of computational modeling approaches have been applied to wide-ranging levels of organization—from molecules to humans. The processes that are modeled include molecular interactions, signaling pathways, metabolic pathways, cellular growth, anatomical structures, and physiological processes. Accordingly, computational approaches differ widely with application.

In this review, we discuss the relevance of current and future applications of computational modeling in nutrition research. To this end, we first introduce important concepts in nutrition and the typical issues for modeling that arise in this field. Then, we give a broader review of some representative modeling approaches that have successfully addressed key nutritional questions. We then proceed to identify knowledge and technology gaps and suggest how the computational approaches may be integrated and extended to address these gaps and bring nutritional systems biology modeling an important step forward in the near future.

### Nutrition and Modeling

Nutrition research investigates the processes by which the living organism receives and utilizes the materials necessary for the maintenance of life and health (as defined by James S. McLester in his classic 1927 textbook) [1]. Traditionally, nutritional research investigates these processes at the level of the whole organism. From a thermodynamic viewpoint, all living organisms exist in a state that is far from equilibrium. To maintain this state, it is of central importance to harvest sufficient energy from the surroundings. This energy comes from the controlled combustion of the macronutrients carbohydrate, fat, and protein. The overarching organizing principle expressed in the Dynamic Energy Budget theory [2], which considers that energy from food is extracted (by the gut), stored in reserves (body fat, protein, and carbohydrate pools), and distributed throughout the body to fuel the processes essential for life. These processes include the generation of heat, maintenance of gradients across cell membranes, the production of gametes, the synthesis of structural mass, the establishment of maturity, somatic maintenance, and maturity maintenance. This organization effectively decouples the organism's internal energy from the external world, facilitating homeostasis. As such this principle has a clear relevance for nutritional physiology.

In contrast to the dietary macronutrient energy sources (i.e., protein, carbohydrate, and fat), dietary micronutrients, notably mineral elements and vitamins, also play a key role for the overall health of the organism. Inadequate amounts of some dietary micronutrients have been demonstrated to cause classic deficiency diseases such as scurvy, beriberi, anemia, goiter, and cretinism. As a third class, various essential nutrients exist that can be used for both energy harvesting, synthesis of structural mass, as well as precursors of specific bioactive compounds. These nutrients include the essential amino acids and the essential omega-3 and omega-6 fatty acids.

Many health disorders are not necessarily caused by dietary deficiencies, but more generally from imbalances between intake and utilization of nutrients. While there is general consensus that proper nutrition can prevent various chronic diseases, understanding the health effects of specific nutritional compounds is extraordinarily complicated. First, delivery of a nutritional perturbation is difficult to control over long time periods and such perturbations often have relatively subtle effects over the time scales typically investigated (as compared to pharmacological compounds whose effects are detected on time scales from minutes to days or weeks). Second, it is very difficult to unravel the distinctive bioactivity of a nutritional compound of interest when it is supplied in a background diet containing hundreds of other bioactive components. Third, it can be difficult to assess the bioavailability of the nutrient of interest, especially at the level of specific target organs or cells.

The problem of bioavailability at the whole body level has had a long history of mathematical modeling, specifically for trace elements. Computational kinetic methods were introduced in nutritional sciences along with the use of stable isotopes where the interpretation of the kinetic data required the development of appropriate mathematical models [3]–[6]. Typically, compartmental modeling approaches are used to describe the absorption, distribution, and elimination of a nutrient. Common to most of these models is the high level of aggregation, where the body is adequately described by only a few compartments. Together, these models aim to provide a rational basis for the determination of the nutritional requirements of the body, and for the understanding of differences in requirements (both locally for organs and at the whole body level) for different micronutrients.

While such traditional modeling methods have been very useful, the real challenge for modeling in nutrition is to help understand and rationally manipulate the complex relationship between nutrition and health, which is determined by the integrated multiscale responses to nutrients, ranging from whole body to subcellular levels of organization and over time scales of minutes to years.

This difficulty is apparent from the problems that arise with current efforts to pinpoint the precise role of nutrition in the metabolic syndrome. At the long time scale and whole body level of organization, a prolonged period (∼weeks to years) of consuming more energy than is expended results in the gradual development of obesity and increases one's risk for developing insulin resistance—a hallmark of the metabolic syndrome. The study of insulin resistance has revealed that the function of this hormone at the level of organs and tissues occurs on the time scale of minutes to hours. For example, insulin stimulation of skeletal muscle glucose uptake, inhibition of hepatic glucose output, inhibition of adipose tissue lipolysis, and a host of other physiological effects occur on this time scale. Methods developed to unravel and quantify the molecular mechanisms underlying these effects have shown the involvement of complex intracellular signal transduction pathways, changes of gene expression, modification of enzyme kinetics, and intracellular molecular trafficking. Furthermore, the production of insulin by pancreatic beta cells occurs in response to glucose and amino acids and can be modulated by fatty acids, all of which can clearly be influenced by diet and nutrition. The unique electrophysiological properties of beta cells are influenced by the metabolism of glucose and fatty acids, while the electrical bursting and oscillatory behavior is coupled to insulin secretion on the time scales of seconds to minutes. Thus, understanding how nutrition impacts the mechanisms underlying insulin resistance requires a quantitative analysis and description of a multiscale, highly coupled regulatory network that includes thousands of components, ranging over subcellular to whole body levels of organization and spanning time scales from seconds to years.

Although a conceptual perspective as outlined above can be derived from literature without too much effort, it is extremely difficult to develop an integrated quantitative understanding that spans the entire complexity of the mechanisms involved. In principle, mathematical models offer this capability and therefore are required to more fully understand the physiological basis not only of the metabolic syndrome, but of the role of nutrition in health and disease in general. Without such a quantitative and integrative approach, it is inevitable that one will get lost in the tangle of bubbles and arrows typical of conceptual models and find oneself unable to weigh the relative importance of each component or interaction in determining the overall physiological phenotype.

The field of mathematical modeling in nutrition is very diverse and presently no single mathematical formalism allows one to generate the required integrated quantitative understanding of nutrition as formulated above. Therefore, in developing our vision of what is needed in the coming years, we now review several representative models that have successfully addressed key nutritional questions and together may help point the way to a more integrative modeling approach.

First, we review modeling approaches for processes at the cellular level describing the biochemical processes (i.e., signaling- and metabolic pathways) that operate to convert food ingredients into energy and building blocks for the cell as the fundamental unit of life. Insight into these processes teaches us how metabolism is regulated at its most basic level. Furthermore, modeling at the cellular level provides the entry point to considering the vast quantity and complexity of “-omics” data.

Second, we review the use of metabolic flux analysis (MFA) as a framework for the quantitative analysis of material fluxes within the single cell as well as between different cell populations and organs, up to the whole body level. Thus, MFA forms a natural bridge between different levels of organization and different time scales.

Thirdly, we review compartmental models of lipoprotein metabolism, because lipoproteins are the major mediators of lipid trafficking between organs, and many processes linked with lipids are associated with the metabolic syndrome, which includes cardiovascular diseases, obesity, and insulin resistance, modern plagues in industrialized societies. Finally, we review mathematical models of body weight and composition regulation and the complex relationship with macronutrient metabolism at the whole body level. Modeling at this whole body physiological level demonstrates the importance of considering long time scales that are characteristic of chronic diseases like obesity and metabolic syndrome.

Of course, we cannot cover all areas of modeling in the field of nutrition in this review. For instance, we will not review models of gut-associated processes of nutrient absorption and bacterial conversion of nondigestible food components into such important compounds as short-chain fatty acids (for a review on the latter, see reference [7]). Another important area that we will not review is models of the neuro-hormonal regulation of food intake and metabolism. Nevertheless, the collection of models that we chose to review ensures that we cover process occurring on a vast space–time spectrum, from nanometer to meter and from microseconds to years. Therefore, the four areas of modeling that we discuss provide a sufficiently broad basis for our goal, namely to review the available computational approaches that are key to answering central questions in nutrition and that can serve as a platform for the development of more integrative systems models.

### Cellular Modeling Approaches

Mathematical models of cellular processes can be used to simulate cellular behavior to better understand the complex mechanisms underlying experimental observations. This understanding may relate to specific research questions such as how the system will react to the addition of a certain substrate (nutrient) or the deletion of a gene. Alternatively, cellular models may also address more general issues such as how control is distributed in a complex network. Predictions can subsequently be experimentally tested, and observed deviations from model predictions can help with data interpretation through the process of modifying the model to better represent the true behavior of the cell [8].

To allow such quantitative simulation and prediction, cellular modeling ultimately aims at a detailed, mechanistic description of molecular processes occurring in single cells. Towards this goal, known pathway structures are translated into differential equations, which—after estimation of the unknown parameters from experimental data—can be used for dynamic simulations of a pathway or network behavior. However, such mechanistic modeling approaches are presently only feasible for rather small pathways or networks. Thus, even on the cellular level, there is a gap between kinetic mechanistic models on the one hand and more coarse-grained modeling approaches on the other hand. The latter are larger in scope (i.e., they encompass more modeled components and interactions—up to the genome scale) but describe the interactions between the modeled components with less mechanistic detail. This section provides an overview of the two categories of mathematical modeling approaches that are used to describe processes on a single cell level and mentions application areas.

#### Mechanistically detailed kinetic models

The first type of cellular models describe molecular mechanisms at the cellular level on the basis of ordinary differential equations. These models consist of balance equations describing the dynamic concentration changes of the considered molecules with appropriate rate laws (e.g., mass action or Michaelis-Menten kinetics). In most cases, these models consider only a few dozens of molecules and either focus on metabolic or on signaling processes. Prominent examples of such models include a model of glucose metabolism of the red blood cell [9], and a model for the yeast *Saccharomyces cerevisiae* that mechanistically describes the organism's response to osmotic shock [10]. The latter describes biochemical reactions comprising receptor stimulation, mitogen-activated protein kinase cascade dynamics, gene expression activation, and adaptation of cellular metabolism with a thermodynamic description of volume regulation and osmotic pressure. As such, this model is one of the few kinetic models that describe processes on more than one cellular level. A recent model of the carbon transfers in the hepatic folate cycle [11] is an example of a model directly related to a specific nutrient. More cellular models can be found in dedicated model repositories (www.systems-biology.org, www.biomodels.org).

From such kinetic models, mechanistic insight about the modeled molecular interactions can be obtained by means of numerical simulation and other computational analyses such as metabolic control analysis, which determines how the control of flux is distributed in metabolic networks [12]. One of the major hurdles in the development of these mechanistic models is that significant uncertainties exist in the molecular mechanisms and in the respective model parameter values. Typically, such information is estimated from either kinetic measurements in an isolated in vitro system, or from parameter optimization methods to fit the model to quantitative (and ideally dynamic) measurement data obtained from the in vivo system. Although currently such data are being generated for single cell organisms, model structure and parameter identification challenges remain huge, particularly with models of larger size [13].

#### Large scale, coarse-grained topological network models

The second type of mathematical models are coarse-grained topological network models. These models—denoting static representations of components (nodes) and interactions (links)—describe the interactions between molecular components with less molecular detail than kinetic mechanistic models (and sometimes without any detail), but often include hundreds to thousands of components, up to the full genome scale. The capability to create such models arose in parallel with the capability to sequence and annotate genomes and the advent of high-throughput “-omics” techniques. These models basically represent an organism-specific collection of components and interactions based upon, for example, the genome annotation and on information from the literature. As such, topological models can be first considered as comprehensive collections of the information about a particular network (i.e., components and interactions) within a specific organism. Well-known examples for such topological models are the signaling networks of the epidermal growth factor receptor [14] and the Toll-like receptor [15] or the recently published complete metabolic network of the human cell [16]. The complexity of these models can even be further expanded by including component interaction, physical arrangement, and evolutionary changes as separate additional dimensions [17].

A special class of topological models, stoichiometric metabolic network models, describes an organism's complete set of metabolic reactions. Typically, stoichiometric models describe the chemical stoichiometries of the biochemical reactions of an organism in its entirety to predict the steady state fluxes of all pathways in the network given the uptake rates of one or more substrates (e.g., nutrients). Such models have been developed for many organisms, including yeast [18], mouse [19], and human [16],[20].

#### Utility of cellular models

Mechanistically detailed kinetic models allow one to numerically simulate the behavior of a small part of the cellular system in response to changes of environmental parameters (e.g., model inputs) or when specific cellular components are modified (e.g., gene knock-outs). Coarse-grained topological network models can be used for qualitative simulations, even at the basic level of curated knowledge. For example, assuming that individual regulatory interactions are either on or off generates a kind of discrete network dynamics much the same as in logical electronic circuits, hence they are referred to as Boolean network models [21]. Surprisingly (because no mechanistic detail was included), patterns in the regulatory properties of such networks often match those found experimentally in the modeled biological system.

Current research tries to uncover and exploit pattern structures in the interactions that make up the topological model so as to infer specific regulatory properties of the network. For instance, metabolic reactions from the recently reconstructed human metabolic network can be positioned in a variant of the so-called bow-tie structure (one that makes a network flexible and robust at the same time [22]) depending on whether or not they are fuelled by essential nutrients [16]. As another example, since metabolic networks appear to be organized in a modular and hierarchical manner, methods for rational decomposition of the metabolic network into relatively independent functional subsets can help us better understand the modularity and organization principle of large-scale, genome-wide networks [23].

Secondly, graphical representations of topological network models may be used to map results of transcriptomics, metabolomics, or proteomic experiments that compare cellular behavior under different conditions. As such, these models allow for a more or less direct linkage between wet lab and model at the -omics level. For instance, representing increased/decreased concentrations in a network context often allows one to efficiently locate by visual inspection the spots in the respective network where most of the changes occurred. Recently, a graph-based algorithm has been proposed that allows one to computationally map transcript data onto a genome-scale metabolic network model. This approach identifies so-called reporter metabolites (i.e., metabolites around which the most significant transcriptional changes occur) from gene expression data as demonstrated by a study on different carbon sources and/or genetic perturbations in yeast [24]. In another approach, a stoichiometric metabolic network model was used to predict putative active regulatory sites in metabolism on the basis of quantitative metabolome data and a thermodynamics-based computational approach [25],[26].

Finally, the concept of constraint-based modeling [27] (an example of which is flux balance analysis) was developed to perform computational simulations with stoichiometric metabolic network models. Such simulations are based on a numerical optimization of a certain biological objective (e.g., biomass yield) within the constraints defined by the steady-state mass balance equations describing the reaction stoichiometry along with energy [28], thermodynamic [25],[26], or physico-chemical considerations [29]. Within certain limits, constraint-based models can predict the effect of metabolic gene deletions on the fitness of a cell and the modulation of phenotypes in response to substrate (i.e., nutrient) availability can be studied in silico and verified experimentally.

#### Towards closing the gap between the two cellular modeling approaches

The future challenge for modeling processes on the cellular level will be to describe larger networks in a mechanistic way. For example, ideally we would like to predict the effects of simultaneous application of a nutrient with a drug such that the best cellular marker for a given response (e.g., hepatocyte insulin response) can be identified. To meet this challenge, models are required that integrate the kinetic as well as the topological approaches. Such models should first be calibrated using experimental flux and “omics” data taken from a set of individuals with well-chosen biological variability (i.e., genetic difference), then used for prediction. On a small-scale, this integrative approach was demonstrated to work for lysine production in *Corynebacterium glutamicum* [30], where various genes were deleted or overexpressed and their effects were correctly predicted. It may also be feasible to construct large-scale mechanistic models by combining mechanistically detailed kinetic models and coarse-grained topological network models [31]. In fact, it was recently shown that complex system behavior is often largely defined by the model structure (i.e., the interaction topology) [32], [33]. This finding further supports the expectation that in order to obtain meaningful predictions most likely only a few molecular processes need to be described in great detail with precise parameters estimates, while the rest of the system can be described using the coarse-grained interaction topology. Therefore, since genome-scale topological network models are now available from microbe to man and high-throughput experimental data are becoming more and more available, it is possible that genome-scale kinetic models can be built in the not-too-distant future [34]. Thus, we foresee that the activities in the field of cellular modeling will eventually lead to a situation where in silico prediction of the effects of nutritional and pharmacological treatments in health and disease will be part of the biological research routine [8].

### MFA

Flux balance analysis is closely linked to an experimental technique called MFA, which allows one to quantify intracellular metabolic fluxes on the basis of acquired experimental data on the uptake and/or production rates of a few metabolites. Stoichiometric metabolic network models, as discussed above in Cellular Modeling Approaches, provide the basic modeling background for MFA [35].

The power of MFA can been significantly augmented by also including experimental data from stable isotope labeling experiments [36]. Metabolic networks are thereby probed with complex mixtures of multiply stable isotope-labeled precursors, and the data analysis proceeds via isotopomer distribution modeling, which is again built around stoichiometric mass balance (or, rather, isotopomer balance) equations [37]–[39]. Stable isotope-aided MFA has found broad application in metabolic engineering efforts aimed at the targeted improvement of microbial fermentations [38]. MFA is increasingly being used in biomedical studies, especially since some investigators have argued that metabolic pathways, rather than genes or proteins, are the true units of function in biology and biochemistry [40]. Thus, flux analysis of target proteins and amino acids in lipoproteins, or of isotope-labeled target compounds in lipoproteins such as glycerol, may help elucidate the mechanisms by which components in the diet (especially dietary fatty acids) affect lipoprotein metabolism and thereby influence the risk of cardiovascular disease (see Modeling of Lipid Transport, below). Furthermore, intrahepatic flux analysis of triglycerides, glycerol, and fatty acids may help elucidate the mechanisms by which components in the diet affect development of hepatic steatosis and subsequent changes in lipoprotein metabolism, which are major contributors to the development of insulin resistance.

Because metabolic fluxes are closely associated with the physiological phenotype, whereas genome-wide stoichiometric models provide the basis for their modeling, MFA bridges the gap between genotype and phenotype, and provides a key for integration of the modeling levels. Indeed, MFA has become a key technique to advance the understanding of biochemical control and gene function. Mapping the effects of gene overexpression and deletion onto changes in intracellular metabolic fluxes, has often revealed unexpected compensatory regulation mechanisms that result in an absence of any clear phenotype. Alternatively, such analyses can help explain an unexpected phenotype. The series of MFA studies on amino acid overproduction by the bacterium *Corynebacterium glutamicum* (reviewed in [41]) provides a number of interesting examples that illustrate this point. One particularly interesting case shows how this organism adapts to growth on acetate instead of glucose. Not only does it activate the classical acetate-induced glyoxylate pathway, but it also greatly increases the amount of oxaloacetate that is decarboxylated to (phosphoenol) pyruvate thereby creating the required acetyl-coenzyme A needed as energy substrate for the citric acid cycle. In mammalian systems, modern genetic techniques can generate highly selective genetic perturbations, targeted globally or to specific organs, which has led to a variety of mouse models with interesting metabolic phenotypes. MFA of these mouse models promises to help elucidate mechanisms of metabolic regulation in both health and disease.

### Modeling of Lipid Transport

Modeling can help to explain the mechanisms involved in the dyslipidemia that occurs with type 2 diabetes, obesity, and the metabolic syndrome. Lipids are a major source of energy and are essential for many processes in the cell, including signaling. Lipids are stored and transported as nonpolar, inert triglycerides. However, neutral lipids are hydrophobic and need to be packed into hydrophilic particles, called lipoproteins, in order to be transported in the blood or into lipid droplets to be stored inside the cell. Lipids are safely stored inside adipocytes in the adipose tissue, however when the lipid load exceeds the adipose tissue storage capacity, lipids are instead stored in other organs resulting in ectopic lipid distribution. These organs, such as liver, heart, pancreas, and muscle are apparently suboptimally equipped to store lipids, and insulin resistance develops. This is obvious in patients with lipidystrophy, who lack adipose tissue and develop severe insulin resistance as well as other symptoms normally associated with obesity [42].

The liver plays a central role in lipid metabolism as it redistributes dietary, systemic (released from adipose tissue), and de novo synthesized lipids into very low density lipoproteins (VLDLs), the precursor for low density lipoproteins (LDLs). In humans, LDL is the major cholesterol carrier in the blood. Under atherogenic conditions it can enter the arterial wall and cause atherosclerosis. The associated dysregulation of lipoprotein metabolism leads to dyslipidemia, which is typically observed in type 2 diabetes, obesity, and the metabolic syndrome [43], explaining why VLDL and LDL have been intensively studied.

The lipoproteins consist of a core of nonpolar lipids, such as triglycerides and cholesterol esters, surrounded by an amphipathic monolayer of phospholipids and free cholesterol. On the surface different proteins (apolipoproteins) are attached, hence the name lipoproteins. Traditionally, the lipoproteins are divided into different classes depending on their protein content and their density, measured by ultracentrifugation. In general, a large particle has a higher lipid to protein content and thus has a lower density.

**Figure 1:**
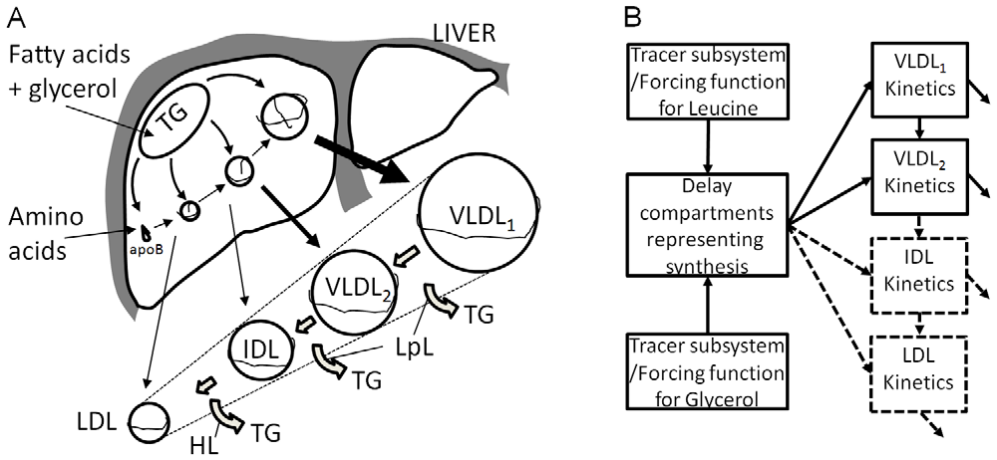
Lipid transport in the body: modeling of apoB100-containing lipoproteins. (A) apoB100 carrying lipoproteins are synthesized in the liver by stepwise addition of lipids to the growing particle. Once secreted, lipoprotein lipase (LpL) and hepatic lipase (HL) may hydrolyze the triglycerides. Intermediate- and low-density lipoproteins (IDLs and LDLs) may be taken up by the LDL receptor. (B) The outline of compartmental models describing lipoprotein kinetics consists of subsystems of tracer molecules (e.g., leucine and/or glycerol), which can be replaced by forcing functions from sample data. A time delay represents the incorporation of the tracer molecules into proteins and triglycerides and is modeled as a series of compartments. The complexity of the blocks representing VLDL_1_, VLDL_2_ (and IDL and LDL) varies with the studied individuals, the length of the study, and the infusion (bolus or primed constant).

The metabolism of the apolipoprotein B100 (apoB100) carrying lipoproteins (VLDL, intermediate density lipoprotein [IDL], and LDL) is briefly outlined in Figure 1A. The kinetic properties of a lipoprotein particle depend on the composition of the different apolipoproteins, the size, and most likely other factors such as the composition of the surface phospholipids. For instance, triglycerides in VLDL are hydrolyzed by lipoprotein lipase, which in turn is activated by apolipoprotein CII on the surface but inhibited by apolipoprotein CIII.

#### Current models of in vivo lipoprotein metabolism provide lipolytic rates

Today, lipoprotein kinetics are studied using infusion of stable isotope-labeled amino acids and glycerol [44],[45]. The enrichment of the stable isotopes is measured for time periods of 8 h up to 5 d in different lipoprotein classes separated by ultracentrifugation.

The main choice of mathematical model to analyze the resulting data has for the past few decades been multicompartmental modeling. Generally these models consist of blocks as described in Figure 1B. A block may contain several compartments, each of which represents material with homogenous kinetics, often corresponding to a separated density fraction of lipoproteins. Recently a model was published where stable isotopes of leucine and glycerol are used as tracers, and VLDL_1_ and VLDL_2_ are modeled in a combined model [46]. In this model each compartment in the VLDL blocks (Figure 1B) corresponds to a specific triglyceride:apoB100 ratio (triglycerides per particle) and is represented by one apoB100 compartment and one triglyceride compartment. The equation for the rate of change of an apoB100 compartment is thus linked to the rate of change of the corresponding triglyceride compartment size. This procedure of tying together the apoB100 and triglyceride models enhances the precision of the model as a whole. As each particle contains one single copy of apoB100, the model provides an estimate of the lipolytic rates (the loss of triglycerides per time unit), which can then be used as a physiological readout for answering study questions related to dyslipidemia.

#### Novel modeling approaches help to link up with molecular mechanisms

There are also new, mechanistically driven approaches emerging in cholesterol modeling. A particle-centered model approach has been described independently by Hübner [47] and by van Schalkwijk [48]. They showed that cholesterol plasma levels can be simulated as resulting from a steady state of a particle distribution. Formulated as a stochastic particle population model, a large number of individual lipoprotein particles must be modeled for a simulation, and considerable computational power is required. Alternatively, as in [48] a large number of lipoprotein compartments similar to the ones figuring in traditional lipoprotein models (see above) may be defined and simulated, thereby greatly improving the computational efficiency. Pearson and coworkers [49] have published a deterministic model for lipoprotein endocytosis in which different processes for the uptake of LDL and VLDL particles and the receptor kinetics are integrated in a model and compared to in vitro data. These model approaches are still in early development, but show that there is a progress in integrating molecular information in the field of lipoprotein/cholesterol modeling, further helping to tie down phenomenological/physiological observations to underlying molecular mechanisms.

#### What can we learn about normal and patho-physiology from models of lipoprotein metabolism?

As shown, stable isotope studies and mathematical modeling provide a tool for the in vivo probing of lipoprotein kinetics and help to reveal mechanisms involved in dyslipidemia observed in various disease states. Results from recent kinetic studies in individuals with the metabolic syndrome have been reviewed recently [50],[51]. These and other results testify that models have played a key role in elucidating the regulation of secretion of the differently sized lipoproteins. For instance using the above model it was shown that type 2 diabetes patients oversecrete the largest VLDL_1_ particles whereas VLDL_2_ production is comparable [51] adding to the knowledge that VLDL is oversecreted in obesity and type 2 diabetes [52],[53]. Moreover, the approach allowed pinpointing liver fat as the best determinant of VLDL_1_ production [54] and of the dynamic response to insulin [55].

#### What is the lipid-lowering mechanism of statins/omega-3/weight loss?

By quantifying lipoprotein metabolism in treated and nontreated individuals, the effect of interventions on lipid metabolism can be studied. Both weight loss and omega-3 treatment have thus been shown to act on lowering plasma lipids by decreasing the secretion [56]–[59], whereas an increased clearance rate was found to be the resultant of the lipid-lowering drugs statins [60].

To elucidate further details of the molecular processes involved in lipid metabolism, in vitro studies are required. The information gained in these molecular studies can serve as scaffolds for models that can then be tested in vivo using the appropriate experimental labeling techniques, analysis protocols, and mathematical models. Clearly, in vivo studies, in combination with mathematical modeling, are essential to provide information regarding both normal physiology, dys-regulation in disease states, and mechanistic insights of drug effects, because this cannot be learned from molecular studies in vitro.

### Modeling Whole Body Metabolism and Body Weight Change

Understanding the dynamics of human body weight change has important consequences for nutrition-related conditions such as obesity, starvation, and wasting syndromes such as anorexia nervosa and cancer cachexia. But making quantitative predictions of body weight and composition changes has proved difficult because of the number of interacting components and the inherent nonlinearity of the system. However several recent mathematical models have substantially improved our ability to integrate whole body metabolism data with body composition data and make quantitative predictions as reviewed below.

#### What proportion of weight loss is attributable to reduced body fat?

**Figure 2:**
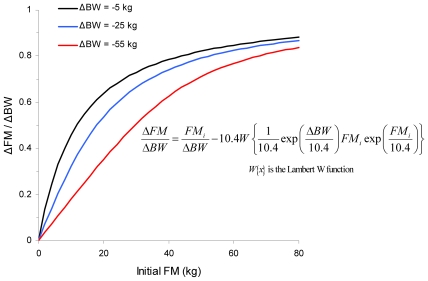
The proportion of body weight loss (ΔBW) from loss of body fat mass (ΔFM) as a function of initial fat mass (FM) for different degrees of weight loss ΔBW as calculated using a novel mathematical model [**62**] **that revisits Forbes classical theory** [**61**] **.**

Ideally, weight loss interventions would primarily result in body fat loss, but unfortunately lean tissue mass is also reduced. A recent mathematical model was developed to quantify the factors that determine the proportion of weight loss coming from body fat versus lean tissue. The basis for the model was a classic theory of Gilbert Forbes who hypothesized that longitudinal body composition changes are described by movement along a logarithmic curve relating lean body mass to fat mass [61]. The new updated equation accounted for the effects of the initial body fat mass as well as the direction and magnitude of weight change as depicted in Figure 2 showing the proportion of weight loss from body fat mass as a function of initial fat mass for different degrees of weight loss [62].

The predictions of the new equation compared favorably with data from human under-feeding and over-feeding experiments and accounted for previously unexplained trends in the data. For large weight changes, such as the massive weight losses found in obese patients following bariatric surgery, Forbes's original equation consistently underestimated the lean tissue loss—a potentially dangerous result. Because the new equation accounted for the magnitude of the weight loss, it provided better predictions of the body composition changes observed in bariatric surgery patients.

#### What is the required energy deficit per unit weight loss?

Weight loss is caused by eating fewer calories than are expended to perform physical work and maintain life. But how many calories translate to one kg of body weight change and what are the biological determinants of this calorie-to-weight loss conversion? The ubiquitous dieting rule “3,500 kcal to lose one pound ” has been used for more than half a century to estimate expected weight loss. Despite its popularity, the biological basis of this rule has been mysterious. A recent mathematical model showed that the caloric equivalent of lost weight is not a constant but rather depends nonlinearly on initial body fat mass, with fatter people requiring a greater energy deficit than lean people for the same amount of weight loss [63]. The magnitude of weight loss also plays a role in determining the caloric equivalent of lost weight and the popular dieting rule was found to be approximately valid only for obese people with initial body fat above 30 kg.

#### What permanent lifestyle changes are required for weight-loss maintenance?

Diet and exercise can successfully cause significant weight loss in obese individuals, but most people eventually regain their lost weight. Weight regain is likely due to a return to the former lifestyle and it is unclear what permanent changes would be required to maintain lost weight. In other words, if an obese person wishes to achieve a specified goal weight then how would their diet or physical activity have to permanently change to maintain their goal weight? A quantitative answer to this question at the outset of an obesity intervention could help both the patient and physician assess whether long-term adherence to the calculated lifestyle change is a realistic proposition. Before a recent mathematical model was developed to address this important topic, such a calculation was not possible.

The mathematical model accounted for the decreased energy requirements at a reduced body weight and incorporates the nonlinear relationship between body fat and lean mass changes [64]. The model calculated the expected change of steady-state body weight loss arising from given changes of dietary energy intake and physical activity. Conversely, the model equations were also solved for the energy intake change required to maintain a particular body weight loss. The model was developed using data from eight longitudinal weight loss studies representing 157 participants with initial body weights ranging from 68–160 kg and stable weight changes between −7 and −54 kg. The model provided the first realistic calculations of body weight and composition change as well as the dietary modifications required for weight loss maintenance. Importantly, the model was implemented using standard spreadsheet software and can therefore be widely used by physicians and weight management professionals [64].

#### Can weight loss interventions specifically target “belly fat”?

The anatomical location of body fat storage is another important issue of body composition. A common question is whether there are ways to target the reduction of fat in specific areas of the body. In particular, it would be desirable to target visceral adipose tissue, commonly called “belly fat,” since fat storage in this area is believed to confer greater risk of cardio-metabolic disease. But what determines the relative change of fat storage in some locations compared with others? Is it possible to “spot reduce” belly fat with certain diet or exercise programs?

A large number of clinical studies have investigated whether diet interventions, exercise, or bariatric surgery can preferentially target the reduction of belly fat, with some investigators concluding that exercise specifically targets visceral adipose tissue. However, a recent mathematical modeling analysis of these data found that changes of visceral adipose tissue do not depend on the type of weight loss intervention. Rather, the model showed that a simple allometric equation with a single parameter explained more than 70% of the variability of the data relating the changes of visceral adipose tissue to changes of overall body fat [65]. The model showed that changes of visceral adipose tissue are primarily determined by overall body fat changes as well as the initial ratio of visceral to total body fat—a variable that also accounted for the influence of sex. The model also correctly predicted how increasing weight loss decreases the proportion of fat loss from visceral versus subcutaneous tissue [66]. The simple allometric equation has clinical utility because it can be used as the appropriate null hypothesis to test whether an intervention specifically targets the reduction of visceral adipose tissue.

#### How does the body decide what fuel mixture to burn?

The food we eat has three macronutrients that the body can use to provide energy: carbohydrate, fat, and protein. But how does the body decide what fuel mixture to use? The composition of our diet clearly plays a strong role, but does our body composition also provide feedback that influences fuel selection? How does fuel selection change during under-feeding or over-feeding?

A two-dimensional ordinary differential equation model of human macronutrient balance was recently developed where the dynamics of the model were constrained to obey the Forbes logarithmic body composition curve [67]. This procedure resulted in a single equation that, for the first time, explained how interactions of diet, energy expenditure, and fat oxidation are quantitatively connected to changes of body composition [68]. Remarkably, the equation (containing no free parameters) accurately predicted the observed changes of body composition and fuel selection during both experimental under- and over-feeding in adult humans when the measured food intake and total energy expenditure were provided as inputs to the model. A similar approach was also used to calculate fuel selection during normal human infant growth and provided the first dynamic picture of how metabolism adapts in concert with changes of diet and energy expenditure to give rise to normal tissue deposition over the first 2 y of life [69].

To better understand the complex interactions among metabolic fluxes contributing to whole body fuel selection, a detailed computational model of human macronutrient metabolism was developed [70]. The model quantitatively tracks the metabolism of all three dietary macronutrients and their interactions within the human body. In particular, the model describes how diet perturbations result in adaptations of whole body energy expenditure, fuel selection, and various metabolic fluxes (e.g., lipolysis, lipogenesis, gluconeogenesis, ketogenesis, protein turnover, etc.) that ultimately give rise to changes of body weight and composition on a time scale of days to years. The nonlinear differential equation model was developed using published human data from over 50 experimental studies and has been subsequently validated using a wide variety of clinical data where the food intake was controlled, including studies of over-feeding, under-feeding, and isocaloric exchange of dietary macronutrients in lean and obese men and women.

The computational model was designed with the specific goal of helping to design, predict, and analyze the results of prospective clinical studies and has been used to identify knowledge gaps and thereby design a novel clinical research protocol currently enrolling participants at the National Institutes of Health (NIH) Clinical Center (ClinicalTrials.gov identifier NCT00846040). Model simulations were instrumental for the design of the clinical protocol to address questions about the length of time required to detect a significant effect during the controlled diet intervention as well as the required magnitude of the intervention and expected sensitivity to interindividual participant differences and uncertainties in measured parameters.

#### What is the cause of involuntary weight loss in patients with advanced cancer?

Often, patients with advanced cancer experience debilitating involuntary weight loss. This wasting condition, called cancer cachexia, is associated with a variety of metabolic changes that affect macronutrient and energy balance. A computational model of macronutrient balance was recently used to integrate the available data on the metabolic changes in patients with cancer cachexia. The resulting computer simulations showed how the known metabolic derangements (e.g., increased proteolysis, lipolysis, and gluconeogenesis) synergize with reduced energy intake to result in a progressive loss of body weight, fat mass, and lean tissue [71]. The model was also used to quantify the contribution of hepatomegaly to the elevated metabolic rate observed in patients with advanced colon cancer [72]. The computational model also provides a new tool to help predict the effects of potential therapies. The model showed that the recently suggested therapeutic approach of lipolysis inhibition could be detrimental to cachexic patients because the simulations predicted that fat preservation comes at the expense of further deterioration of lean body mass due to increased reliance on body protein for oxidation and gluconeogenesis. Importantly, the model helps elucidate the mechanisms of body weight change in this complex and serious disorder where it would be prohibitively difficult and invasive to attempt a comprehensive clinical study.

## Discussion

Here we will put the available modeling approaches into perspective and work towards an approach for fully integrated systems biology modeling applications in nutritional sciences.

### 

#### Classification according to space–time scales

Classification and dimensions play a crucial role in comparing and assessing the different model approaches. An excellent introduction on different levels of mathematical modeling, especially on multiscale modeling in biology, by Southern et al. [73] addresses this point. In their publication, the authors divide the model universe into 11 categories, according to levels of biological organization. These range from the smallest scale at the quantum level to the largest scale at the environment level.

**Figure 3:**
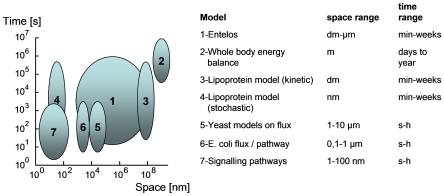
Overview of the time–space range of the diverse model types, which are discussed in this review.

Following this classification scheme, the nutritional models discussed in this review span seven levels, from macromolecular to organism. Armed with such a hierarchical view, one can also distinguish between single level models, which are confined to one level of organization, and multilevel models that span two or more levels. In fact, to date, there are only a few examples of multilevel models in biology. The most famous is the Virtual Heart, developed by a large consortium formed along the Physiome Project [74],[75]. Another example is provided by the PhysioLab platforms developed by Entelos to predict the efficacy and safety of therapeutic interventions in areas such as metabolism, rheumatoid arthritis, and atherosclerosis [76],[77]. To visualize their level and range, and for discussion purposes, we have positioned in Figure 3 an Entelos-type multilevel model together with the model approaches presented in this review in a quantitative context. In addition to the space dimension given by Southern, we also added the time scale dimension of the models, as some approaches at the same organizational level differ significantly with respect to their characteristic time scale. Clearly, the Entelos model approach is the most advanced multilevel approach among the given models. It can also be appreciated from Figure 3 how far a signal transduction model is removed from a whole body model. This does not mean that a connection is a priori impossible, but simply that in order to link these two models one has to bridge numerous levels of time and space.

At this point we can ask the question, what class of models should a nutritional scientist choose for her/his research? It is clear that there is no right or wrong answer to this question, but each modeling project should be designed fit the research questions it should help to answer. Thus, a model constrained to a single level is best suited to quickly integrate experimental data of that same dimension. This helps to initiate and stimulate a collaboration between wet lab and dry lab researchers, because results of such a model can provide rapid feedback and develop new insights that will lead to improved experimental designs. The models of metabolism and body composition change discussed in this paper are very good examples. Interestingly, these minimally parameterized models can predict endpoints of body composition and energy metabolism reasonably well on time scales ranging from days to years. An important conclusion from this observation is that these models may provide ideal constraints and a framework to embed smaller functional units. Working single level models can be established in a matter of weeks as far as the basic equations are concerned, but the careful parameterization and validation require considerably more time and effort.

The modeling of cholesterol (models 3 and 4 in Figure 3) provides examples for an approach that could be coined as “middle out” approach, where modeling is started from a single, intermediate level and the model is subsequently extended upwards to include more physiological constraints or downwards to integrate molecular processes. Often, such an approach can be helpful to integrate new experimental data within an existing single level modeling framework.

Multilevel models are the most resource-intensive models and will require a high level of ambition. As an example, the PhysioLab platforms developed by Entelos are designed to reproduce clinical observations in humans, based on detailed mechanistic descriptions of processes from the cellular to the organism level. Rather than attempting to include all aspects of physiology, or all intracellular events, the design process identifies the key biological processes and molecular players that are necessary to answer the clinically relevant questions. Data are gathered from the literature to quantitatively describe these processes by coupled ordinary differential equations, which are solved numerically as a function of time. After calibrating the model at various levels to be consistent with known in vitro and physiological data, the final model predictions are validated against clinical trial results for several existing therapies. While these modeling platforms are developed for commercial purposes in the field of pharmaceutical research and development, the approach may also be suitable for a range of multilevel problems in nutritional science and is likely better suited to producing physiologically meaningful results than direct attempts to extend detailed models at the signaling level “upward” to the multicellular context.

#### A lack of overlap between existing models at different space–time scales is a hurdle for the integration of existing models

**Figure 4:**
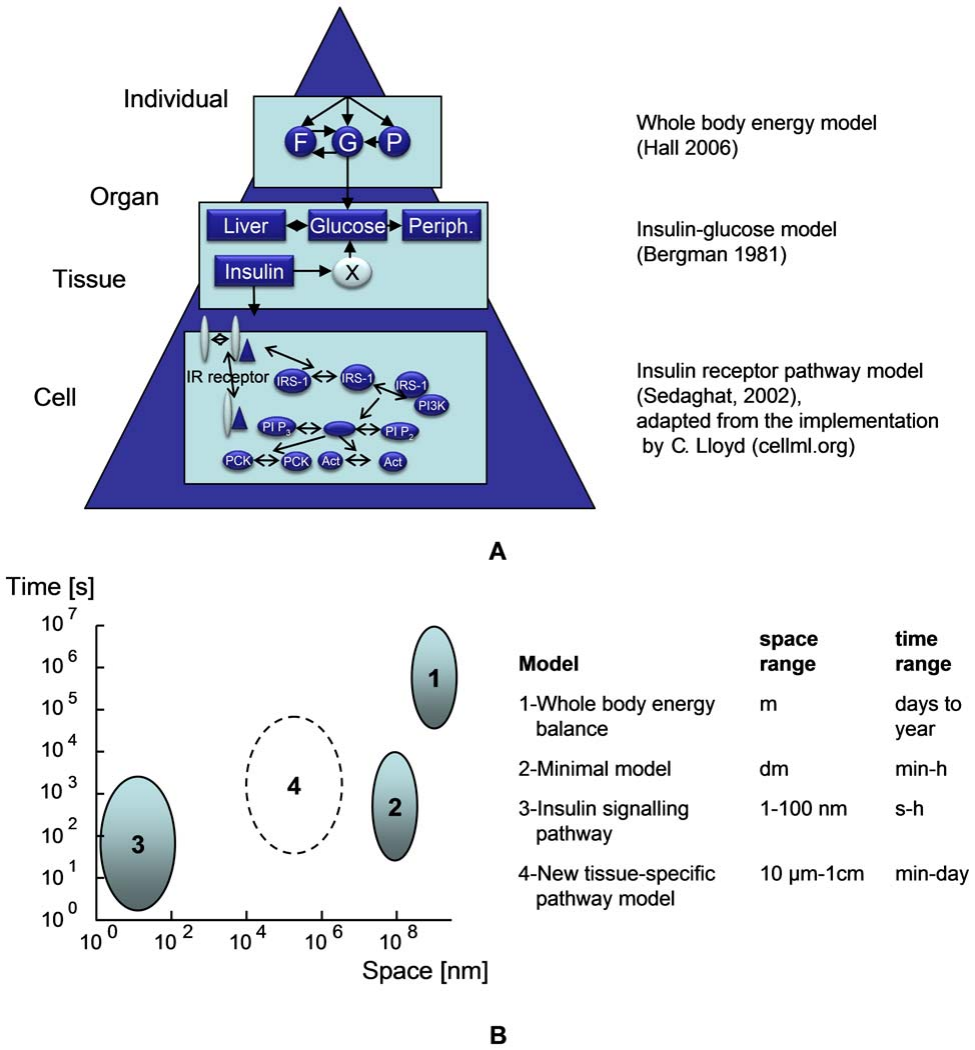
Example of a future multiscale model in the area of insulin resistance, built from three existing models [78]–[80]. (A) schematic overview of the different model layers. (B) Individual model layers plotted along their time–space dimensions. Model 4 denotes a new model that enables the incorporation of tissue-specific gene expression data, which form an important data source from the nutritional wet lab.

There are several areas in nutritional research (e.g., metabolic syndrome, weight management, or degenerative diseases) that provide interesting candidates for building multiscale models. To illustrate the issues involved, we return to the example of insulin resistance as a central issue in nutrition-related disease. In Figure 4A, we have tried to construct an example of a multiscale model for the disease process of insulin resistance by using existing models. On the top level, the organism level, a body weight dynamics model [61],[78] accounts for overall energy and macronutrient balance of the body. This model has been calibrated using data from controlled diet studies that measured energy expenditure, respiratory quotient, oxidation rates of fat, protein, and carbohydrates in conjunction with body weight and composition changes. On a lower level, an insulin-glucose model links changes in glucose and insulin levels. Such a model (e.g., the “minimal model” of glucose metabolism [79]) uses plasma data from glucose tolerance tests or glucose-insulin clamps. This model is coupled with the top level model via the carbohydrate energy flows. At the lowest level, a subcellular model of the insulin signaling pathway [80] simulates the response of pathways affecting glucose, protein, and fat metabolism resulting from changing insulin concentrations. Signaling pathway models are typically calibrated using data from in vitro cell systems and in vitro enzyme kinetics. Such a multilevel model would be able to connect insulin action at a cellular level with insulin sensitivity represented by the minimal model and resulting in shifts of whole body energy metabolism and body composition.

However, analyzing this model in the time–space plot (Figure 4B), one can see a large gap between models 2 and 3 representing the gap between physiological modeling and cellular modeling. We believe this to be a general phenomenon in nutrition: knowledge and models with a high level of detail at the cellular level, a physiological model spanning the levels of organ to whole body that include the main regulatory mechanisms, and a picture of general trends in resulting phenotypes that have been obtained from nutritional intervention studies and/or statistical population analyses. What is missing is a middle-out model from the micrometer to centimeter range, which covers processes between cells, and within and between tissues on a time scale from minutes to days. However, it is also apparent from Figure 4B that models targeted to one level can be used to inform and constrain models at differing levels. To illustrate this point, the model that predicts the proportion of weight loss from body fat as a function of initial fat mass for different degrees of weight loss (Figure 2) [81] has been used to provide a quantitative constraint on how the body regulates metabolism of carbohydrate, fat, and protein [61]. This information was useful for the development of a detailed computational model [78] that simulates the physiological mechanisms of fuel selection at the whole body level underlying the regulation of body composition change. The next step requires adding further mechanistic detail at smaller space–time scales, such as hormonal regulation of nutrient uptake and utilization following meals [82]. Such a process demonstrates a logical path towards generating models with the required level of detail for nutritional research.

#### Experimental data has become increasingly available at the intermediate level between microscopic and macroscopic

Major sources of new insight in nutritional sciences are generated on exactly the level missing in the glucose-insulin example. For example, insights regarding insulin resistance and the metabolic syndrome have been obtained by investigating the function of macrophages in specific tissue [83], by studying the function of adipocytes under various dietary conditions [84], or by characterizing the effect of tissue-specific gene knock-outs on metabolic status [85],[86]. To integrate these experimental data, the initial concept should be extended with a missing fourth model that links gene-specific information from cell systems with physiological processes on the tissue level; this would lead to a functional embedding of cellular information.

In our glucose/insulin example we can develop models for both endogenous glucose production (primarily a liver-based process) and glucose utilization (dominated by neural tissues and the musculature) as measured in response to glucose tolerance tests or glucose-insulin clamps. Details on the molecular mechanisms underlying the physiological responses of each tissue (derived from “-omics” studies) can then be added. Such efforts could help explain how a complex organism such as the human reacts to the dietary stress of, for example, meals with a high glycemic index, or excess saturated fat. A model at this level would also provide a platform for linking up with models of how imbalanced nutrition interacts with processes involved in both metabolic health and diet-induced disease development.

The need for models that can span levels from cells to tissues, organs, and organism is further highlighted by the question of how to translate studies done on humans versus studies done in cell culture. For example, how would one try to connect the results of a study using hepatocytes grown on media with very high saturated fats with a person eating a high saturated fat diet? When one considers that many experimental results obtained using tissue cultures are often extrapolated to humans, any study related to human nutrition requires models that tie the microscopic with the macroscopic level.

#### The middle-out design strategy is a promising approach to generate new multilevel nutritional models

Summarizing the previous discussion, new multilevel nutritional systems biology models are needed that should (a) be complete in a space–time dimension sense and (b) include data and mechanisms to link adjacent levels. For the development of such models, we propose the “middle-out” design strategy, that focuses on the level at which the most experimental data are available, and extends downward as well as upward. Going downward, it is not necessary to include all possible details in the model. Indeed, engineering models used to design integrated circuits or airplanes do not model the condensed matter physics responsible for the electrical properties of the circuit components. Rather, it is important to identify the minimum set of players and mechanisms that are essential to explain the known facts. Going upward, nutritional models should preferably link to endpoints that can be quantified in human intervention studies. Here, it is essential to at least include biomarkers that can play the role of surrogate endpoints. These can later be linked explicitly with true physiological readouts. Having experimental data at two or more levels then allows one to build a mechanistic model that effectively describes the higher aggregation level as a consequence of the phenomena at the lower level, and is consistent with all experimental data. In the Virtual Heart model, data at the cellular level (ion transport) and at the organ level (heart anatomy) thus provide the means to realistically describe cardiac arrhythmias. In the PhysioLab platforms, in vitro data on cellular behavior are synthesized to give an organ level description of, for example, inflammation, that can then be calibrated against clinical observations. Ideally, the model design strategy enables the identification of knowledge gaps: areas where the model is incapable of bridging between the levels thereby indicating that an essential player or mechanism has been left out.

#### Generate phenotypic data to characterize both health and disease states

In many disease conditions, subphenotypes have only been poorly recognized. Model building for nutrition research can only succeed to the extent that phenotypes have been classified and characterized, especially if one is interested in understanding transitions between health and disease.

#### Use the virtual patient concept to deal with variability

One of the central emerging issues in interpreting experimental data is the fact that an enormous natural variation occurs between individuals in any nutritional study. This variability has to be dealt with in nutrition models. In fact, the variation can be readily simulated by a multilevel model because one single-model structure can lead to several physiological outcomes and different phenotypes depending on the lower level states. The latter may be characterized incompletely, thus allowing for the variation of experimental data to be adequately captured by the model. Basically there are two ways of handling this situation: probabilistic modeling, or a deterministic description of “virtual patients” [76],[77]. In the latter, multiple parameterizations of the model are developed, each corresponding to an observed or hypothesized individual patient (or patient group). Model simulations are performed over cohorts of such virtual patients, and the results (phenotypic readouts) can be matched to experimental observations in cohorts of real patients, which are frequently only available as cohort averages.

#### Use biomarkers to bridge microscopic and macroscopic levels

In a multilevel nutritional model, going from the lowest to the highest level in space–time, it does not seem feasible to retain the full mechanistic detail of the lowest level simply because data at the (sub) cellular level will generally not be available. However, we may use the increasingly available “-omics” data to develop biomarkers, or proxies of what is going on at this lowest level.

So far, gene and pathway-directed modeling has been developed in a subcellular to cellular dimension, and kinetic modeling of intracellular phenomena may become more and more routine (see above Cellular Modeling Approaches). At a higher level, integrated genomics, proteomics, and metabolomics data can potentially also be used to bridge between cellular and physiological states and fill the modeling gap between the microscopic and the macroscopic levels. One can envision that through continued and massive experimentation signatures will be developed that are characteristic of specific pathway perturbations or disease states. These signatures can give a description of the state of the transcriptome, proteome, and/or metabolome and thus link to models developed on this level. The challenge here is not the modeling (e.g., “-omics” data have been used in conjunction with pharmacokinetic/pharmacodynamic models [87]), but rather the choice of experiments. Which model systems are most representative of the in vivo situation, which systems are most accessible, and how many signatures can be defined? Nevertheless, it has been shown that cellular signatures can indeed be related successfully to “-omics” data obtained in vivo or ex vivo [88],[89], which demonstrates how model systems can be used in a multilevel approach. On the other hand, the “-omics” signatures are linked to functionally characterized states, such as the outcome of a drug treatment or a disease on a cellular, supracellular (e.g., coculture assays as used by the profiling company Bioseek [90]), or on the organism level. Omics signatures, for example in the form of biomarker patterns, also provide a means to characterize virtual patients and virtual patient cohorts and link with the phenotype at the highest level.

### Summary and Conclusion

From this review and discussion it is apparent that two issues in computational modeling in nutritional sciences now need major attention. First, the complex biological research questions, typical for nutritional sciences, often require a multilevel modeling approach. However, this is a time and resource intensive effort that is best undertaken within a large research consortium. Second, a central need exists for models and experimental data that bridge the microscopic and the macroscopic levels. Most animal disease models or human nutritional studies currently provide tissue-specific “-omics” data, whereas cell–cell interaction data is becoming increasingly available from in vitro systems. To interpret this new hybrid of wet lab data, dedicated computational models are required to deal with cell-specific expression data in a physiological context.

We identified the middle-out strategy as a promising one for generating the required nutritional computational models and the virtual patient concept is a convenient way to deal with the large individual variation typical of nutritional studies. To characterize (sub) groups of individuals, much will be gained by a careful classification and characterization of disease (sub) phenotypes. Finally, the use of biomarker signatures derived from integrated “-omics” data has a potential to bridge the microscopic and macroscopic levels. In conclusion, we have put available computational modeling approaches for nutrition into perspective, and we have suggested the essential elements of an approach for future fully integrated systems biology models for application in nutritional sciences.
